# Antifibrotic Activity of Acylated and Unacylated Ghrelin

**DOI:** 10.1155/2015/385682

**Published:** 2015-04-16

**Authors:** Elia Angelino, Simone Reano, Michele Ferrara, Emanuela Agosti, Andrea Graziani, Nicoletta Filigheddu

**Affiliations:** Department of Translational Medicine, University of Piemonte Orientale, Via Solaroli 17, 28100 Novara, Italy

## Abstract

Fibrosis can affect almost all tissues and organs, it often represents the terminal stage of chronic diseases, and it is regarded as a major health issue for which efficient therapies are needed. Tissue injury, by inducing necrosis/apoptosis, triggers inflammatory response that, in turn, promotes fibroblast activation and pathological deposition of extracellular matrix. Acylated and unacylated ghrelin are the main products of the ghrelin gene. The acylated form, through its receptor GHSR-1a, stimulates appetite and growth hormone (GH) release. Although unacylated ghrelin does not bind or activate GHSR-1a, it shares with the acylated form several biological activities. Ghrelin peptides exhibit anti-inflammatory, antioxidative, and antiapoptotic activities, suggesting that they might represent an efficient approach to prevent or reduce fibrosis. The aim of this review is to summarize the available evidence regarding the effects of acylated and unacylated ghrelin on different pathologies and experimental models in which fibrosis is a predominant characteristic.

## 1. Introduction

Repair of damaged tissues is a complex physiological process that results in the deposition of extracellular matrix (ECM) components by resident fibroblasts [1]. Although the deposition of ECM proteins is normally a transient event, repeated tissue injuries in chronic pathologies or dysregulation of this process can lead to fibrosis and, eventually, to organ dysfunction [2]. Fibrosis can affect almost all tissues and organs, including heart, liver, kidney, lungs, and skin, therefore representing a major health issue for which efficient therapies are needed.

Regardless of the specific fibrotic disease and organs affected, the mechanisms involved in the progression of this pathology are very similar. Indeed, damaged tissue repair can be recapitulated in four overlapping phases, hemostasis, inflammation, proliferation, and remodeling in which several cell types, closely interconnected to each other, play an important role [3]. During the phases of hemostasis and inflammation, platelets secrete cytokines, including platelet-derived growth factor (PDGF) and transforming growth factor-*β* (TGF-*β*) that, in turn, recruit macrophages, neutrophils, and natural-killer cells to the site of injury. These cells, besides removing dead cells, debris, and pathogens, release cytokines that trigger activation and proliferation of resident fibroblasts, thus affecting ECM production [4]. For example, macrophages release TGF-*β*1 that controls a wide spectrum of activities, such as promoting fibroblast differentiation into active myofibroblasts, inducing ECM protein expression [5, 6], and repressing the expression of matrix metalloproteinases (MMPs), key proteins able to degrade several ECM components [7]. In addition, macrophages release tumor necrosis factor-*α* (TNF-*α*) and interleukin-1*β* (IL-1*β*) that promote fibroblast activation and fibrotic tissue deposition [2]. Tissue damage and inflammation increase reactive oxygen species (ROS) production, which, in turn, contributes to fibrosis, enhancing the secretion of fibrogenic factors [8].

Acylated and unacylated ghrelin are circulating peptide hormones encoded by the ghrelin gene which are mainly released from the stomach during fasting [9]. The 117-amino acid preproghrelin undergoes proteolytic cleavages leading to the mature ghrelin peptides and to another biological active peptide named obestatin [10]. The acylated form, through high affinity binding to the growth hormone secretagogue receptor type 1a (GHSR-1a), induces GH release and promotes food intake, adiposity, and positive energy balance [11–13]. Alongside its role in feeding and energy homeostasis, ghrelin exerts also many other biological activities, including cardioprotection and enhancement of cardiac function [14], a strong anti-inflammatory activity [15], antioxidant activity on several cell types and tissues such as liver, heart, and lung [16–19], and neuroprotective activities [20]. The acylated ghrelin anti-inflammatory function mainly depends on its direct effect on T lymphocytes and monocytes, in which it inhibits the expression of proinflammatory cytokines such as IL-1*β*, IL-6, and TNF-*α* [21].

Acylation of ghrelin is essential for its binding to GHSR-1a, since the unacylated form does not activate this receptor, unless administered at very high concentrations, in which case it acts as a functional agonist [22–25]. However, both acylated and unacylated ghrelin share high affinity binding sites in a number of cell lines and tissues, where they mediate several activities, such as protection from apoptosis and oxidative injury [26–32], stimulation of cell differentiation [33–36], induction of proliferation [30, 37–39], and protection of skeletal muscles from wasting [40–42]. These effects suggest the presence of a not yet identified common receptor of both acylated and unacylated ghrelin. In addition, some biological activities are elicited only by the unacylated but not the acylated form of ghrelin, suggesting the existence of a specific receptor for unacylated ghrelin [39, 43–45].

Circulating levels of acylated and unacylated ghrelin are often altered in pathological states associated with fibrosis and this suggests a role for these hormones in tissue homeostasis and/or in etiology of these conditions ([46–52], Table 1).

## 2. Acylated and Unacylated Ghrelin as Antifibrotic Factors

### 2.1. Heart

The massive deposition of collagen in the heart that occurs upon several stimuli, such as cardiomyocyte death, inflammation, hypertension-induced enhanced workload, hypertrophy, or chemotherapy with doxorubicin, plays a crucial role in cardiac remodeling after heart injury and may contribute to ventricular arrhythmias, left ventricular dysfunction, heart failure, and sudden cardiac death [53].

Together with inflammation, cardiac fibroblasts, the most abundant cells in the heart, are the main players in cardiac remodeling: upon injury they undergo proliferation and synthesize collagen to replace the necrotic or apoptotic cardiomyocytes [53].

**Figure 1:**
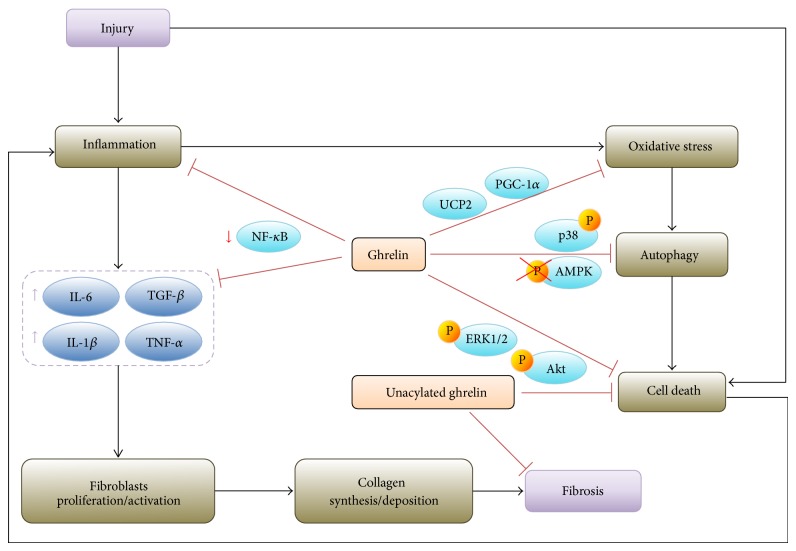
Schematic representation of the molecular pathways involved in the antifibrotic activity of ghrelin and unacylated ghrelin. See text for details.

Due to the antiapoptotic and anti-inflammatory activity of ghrelin, several researchers investigated the antifibrotic effect of acylated and unacylated ghrelin in different models of cardiac injury. Doxorubicin, an antibiotic used in chemotherapy, alters cardiomyocytes energy metabolism and induces their apoptosis, thus determining myocardial fibrosis, which eventually results in cardiomyopathy and congestive heart failure [54]. Accordingly with the in vitro data on the cardioprotective effect of acylated and unacylated ghrelin against doxorubicin-induced apoptosis of cardiomyocytes [26], it has been recently demonstrated that both peptides are effective in inhibiting the cardiotoxicity of this drug also in vivo [55, 56]. Unacylated ghrelin displays antiapoptotic effects on cardiomyocytes through the activation of the prosurvival ERK1/2 and PI3K/Akt signaling pathways ([26, 55], Figure 1). Acylated ghrelin seems to play an important role in the regulation of autophagy, a cellular pathway involved in protein and organelle degradation. Although this cellular pathway is normally a protective mechanism, excessive autophagy can destroy essential cellular components and eventually induce apoptosis [57]. Doxorubicin treatment induces oxidative stress, autophagy, apoptosis, and, finally, cardiac dysfunction and collagen deposition in the heart [56, 57]. In this experimental model of cardiac injury, acylated ghrelin inhibits ROS-induced autophagy and cardiomyocyte death through the inhibition of AMPK and activation of p38-MAPK pathway ([56], Figure 1), thus leading to a decrease of doxorubicin-induced fibrosis and cardiac dysfunction. The antifibrotic effects of acylated and unacylated ghrelin have been demonstrated in other experimental models of cardiac injury, such as isoproterenol administration, myocardial infarction (MI), and spontaneous or diabetes-associated hypertension [58–62]. The subacute injection in rats of the *β*-adrenergic agonist isoproterenol induces myocardial injury and fibrosis and increases myocardial ghrelin expression and plasmatic acylated ghrelin levels [58, 59]. In this model, acylated ghrelin treatment ameliorates myocardial function and reduces fibrosis, although the mechanisms of such a protection have not been elucidated [59]. The unacylated form of the peptide displays similar effects, suggesting that the antifibrotic activity of ghrelin is mediated by both GHSR-dependent and GHSR-independent pathways [59].

Ghrelin has a positive effect on cardiac remodeling and cardiac function also in rats undergoing MI by coronary artery ligation. MI induces a strong increase in tissutal IL-1*β* and TNF-*α* that is inhibited by the chronic administration of ghrelin [60]. Ghrelin also blunts the induction of MMP-2 and MMP-9 that could be viewed as an inhibition of overall fibroblasts activity [60]. However, in spontaneously hypertensive rats, the synthetic GH-secretagogue hexarelin prevents cardiac fibrosis by inducing, rather than by inhibiting, MMP-2 and MMP-9 activity [61]. Notably, unacylated ghrelin, despite reducing cardiac fibrosis in diabetic mice, has no effect on other MMPs involved in cardiac fibrosis development such as MMP-8 and MMP-13 [62]. The effect of unacylated ghrelin treatment was in fact investigated also in db/db diabetic mice compared to nondiabetic mice [62], since cardiac fibrosis is also observed in diabetic patients without hypertension [63]. In this model of diabetic mice, unacylated ghrelin impairs collagen accumulation by upregulating adiponectin cardiac expression [62], which is known to prevent myocardial hypertrophy and fibrosis [64, 65].

### 2.2. Liver

In liver, hepatitis C or B viral infections, autoimmune diseases, alcohol abuse, and nonalcoholic fatty liver disease (NAFLD) can progress to a severe fibrotic disease in which parenchymal tissue is replaced by nonfunctional fibrotic tissue, a condition defined as cirrhosis [66]. Removal of the causative agent, such as viral infections, could revert liver fibrosis, but in the case of autoimmune diseases and NAFLD the causative agent is not clearly defined and the identification of new agents that could modulate this process is of pivotal importance [67].

In patients with alcoholic hepatitis and chronic hepatitis C, plasmatic ghrelin levels are lower than in healthy subjects and negatively correlate with the severity of fibrosis ([48], Table 1). Circulating ghrelin levels also correlate with other hepatic fibrotic diseases; however, in the case of patients with NAFLD, a worsening of the fibrotic stage is associated with high plasmatic concentration of both acylated and unacylated ghrelin ([50], Table 1). Interestingly, a screening of miRNAs expression in visceral adipose tissue of NAFLD patients revealed that miR-132, of which the ghrelin gene is a predicted target, is downregulated in nonalcoholic steatohepatitis (NASH) compared to non-NASH patients [68], although a biological validation of this relationship still needs to be performed.

Although the causative relationship between ghrelin circulating levels and NAFLD is not defined, ghrelin might have a therapeutic potential in this and other hepatic pathologies, as demonstrated in several experimental models. The most used models to induce hepatic fibrosis include CCl_4_ or thioacetamide (TAA) administration to rodents, which lead to oxidative stress-mediated liver cirrhosis [69]. Another model to induce liver fibrosis consists in bile duct ligation (BDL), which causes accumulation of hydrophobic bile acids in the liver, leading to ROS formation, oxidative damage, inflammatory cell accumulation, and the increase of serum proinflammatory cytokines [70]. In addition, NAFLD may be reproduced in rats by feeding animals with a high-fat diet, thus inducing liver fat accumulation, inflammation, and cellular necrosis [71]. In this model, ghrelin treatment blunts the induction of TNF-*α* and IL-6 expression, counteracts hepatic oxidative stress, and inhibits hepatic cell apoptosis [72]. The beneficial effects of ghrelin on liver injury and fibrosis have been pointed out by other studies as well. Indeed, in rats with chronic hepatic fibrosis caused by BDL, ghrelin administration prevents hepatic damage by blunting the BDL-induced increase of TNF-*α*, IL-1*β*, and IL-6 plasma levels [73]. Moreover, ghrelin treatment impairs neutrophil infiltration and diminishes the amount of myofibroblast accumulation in the injured liver [48, 73]. Accordingly, ghrelin downregulates the expression of collagen-*α*1 and TGF-*β*1 in primary hepatic stellate cells (HSC), the main hepatic fibrogenic cells [48], resulting in a diminished collagen deposition [48, 73]. Ghrelin features anti-inflammatory and antifibrotic effects also in TAA-induced hepatic injury in rats where it attenuates liver injury and collagen deposition through inhibition of hepatic cell apoptosis and antioxidative activity, in a way partially mediated by the induction of nitric oxide (NO) [49].

Finally, the physiological role of the ghrelin gene in the establishment of liver fibrosis was investigated exploiting ghrelin knock-out mice that display much more severe CCl_4_-induced liver injury and fibrosis compared to wild type animals, suggesting that endogenous ghrelin is required for a proper response to liver damage [48].

### 2.3. Kidneys

Ghrelin is expressed in kidneys and its expression is altered in pathological conditions such as glomerulopathies, in particular in the proliferative form, in which the immunoexpression of ghrelin is abated [74]. Moreover, the expression of ghrelin negatively correlates with the profibrotic protein endothelin-1 and interstitial inflammatory cell infiltration, suggesting that the loss of ghrelin could contribute to the development of renal interstitial fibrosis, which is the common feature of different end-stage renal diseases [74].

The renin-angiotensin system (RAS) is a well-known regulator of blood pressure and contributes to the development of target organ damage due to hypertension. Angiotensin-II (AngII) is the main mediator of RAS-induced chronic kidney damage through multiple mechanisms, including promotion of inflammation, fibrosis, oxidative stress, and senescence [75]. Indeed, in the experimental model of chronic kidney disease induced by AngII infusion, the kidneys display increased ROS and an accelerated tissue senescence [76, 77]. In addition, treated mice express higher levels of TGF-*β* and plasminogen activator inhibitor-1 (PAI-1) than saline-infused animals [78]. In this model, ghrelin impairs renal tubular damage, fibrosis development, and senescence by both reducing the oxidative stress and maintaining the redox state. This is mediated by the induction of UCP2 and PGC1*α* that affect ROS production and mitochondriogenesis, respectively ([78], Figure 1).

The antifibrogenic activity of ghrelin was demonstrated also in a rat model of renal damage obtained by unilateral ureteral obstruction (UUO), which results in tubular injury and cell death, with interstitial macrophage infiltration [79]. In this model, ghrelin protects renal tubular cells from apoptosis, impairs macrophage infiltration, and reduces the induction of the proinflammatory cytokines IL-1*β*, TNF-*α*, and monocyte chemoattractant protein-1 (MCP-1) [80]. Moreover, this work demonstrates that ghrelin attenuates renal fibrosis by inhibiting fibroblast differentiation and by blocking epithelial mesenchymal transition (EMT), thus stabilizing the epithelial phenotype [80]. The mechanisms through which ghrelin elicits its antifibrotic activity involve the reduction of collagen I/III, fibronectin, and *α*-SMA expression via inhibition of the TGF-*β*1/Smad3 signaling pathway [80].

### 2.4. Lungs

Lung fibrosis occurs as a consequence of acute lung injury leading to persistent respiratory failure. Lung fibrosis is usually differentiated into distinct types, including diffuse fibrosing alveolitis, diffuse interstitial fibrosis, and idiopathic pulmonary fibrosis, which is considered the most common and severe form of pulmonary fibrosis [81]. Currently, there are no therapies to counteract acute lung injury progression and lung transplantation remains the only possible intervention in end-stage disease [81].

Acute lung injury is characterized by the damage of the alveolar capillary barrier, neutrophil accumulation, and the induction of proinflammatory cytokines, followed by devastating lung fibrosis [82, 83]. In particular, the exfoliation of alveolar epithelial cells from alveolar septa leads to the activation of fibroblasts and the subsequent massive ECM deposition [82].

Cecal ligation and puncture (CLP), the most used technique to induce peritonitis and sepsis, also induces lung injury and fibrosis as direct consequence of hypoxemia, neutrophilic inflammation, and alveolar edema [83].

In CLP-treated rats, ghrelin attenuates acute lung injury and mortality through inhibition of nuclear factor- (NF-) *κ*B activity ([84], Figure 1). NF-*κ*B is a transcription factor that regulates gene expression of several cytokines, including TNF-*α*, IL-6, IL-1, and IL-8 [85]. Accordingly, treatment with ghrelin reduces pulmonary levels of TNF-*α* and IL-6 in CLP-treated rats [84].

Another experimental model used to induce acute lung injury in rodents is the intratracheal injection of bleomycin that promotes massive cell death, neutrophil and lymphocyte infiltration, cytokine production, and fibrosis [83, 86]. In bleomycin-treated mice, ghrelin administration improves animal survival in a dose-dependent manner and maintains lung architecture by reducing fibrosis [86]. This antifibrotic activity is due to the impairment of neutrophil infiltration and accumulation in bronchoalveolar lavage fluid and through the inhibition of proinflammatory cytokines and of IGF-1 release, which promotes collagen production by fibroblasts [86]. In addition, the inhibition of alveolar epithelial cell death, observed in ghrelin-treated mice, represents another mechanism that contributes to ghrelin antifibrotic effects, since the prevention of the denudation of alveolar membranes impairs the subsequent fibrosis establishment [86].

In the same model of lung fibrosis, the traditional Japanese herbal medicine rikkunshito, known to stimulate a strong secretion of ghrelin, reduces lung fibrosis and ameliorates the systemic cachectic condition [87]. However, rikkunshito effects are only partially due to the associated ghrelin increase, since it maintains its protective effects also in mice devoid of the ghrelin gene [88].

### 2.5. Systemic Sclerosis

Systemic sclerosis, or scleroderma, is an autoimmune chronic connective tissue disease characterized by extensive fibrosis of the skin and internal organs, including lungs, gastrointestinal tract, kidneys, and heart [52]. Plasmatic levels of acylated and unacylated ghrelin are lower in systemic sclerosis patients than in healthy controls and even lower in patients with interstitial lung disease, suggesting that acylated ghrelin levels inversely correlate with tissue fibrosis ([52], Table 1). Consistently, acylated ghrelin treatment of fibroblasts isolated from systemic sclerosis patients reduces TGF-*β*1 expression and collagen production [52].

Skin scleroderma might be experimentally induced in mice by subcutaneous injections of bleomycin that result in increased dermal thickness, a higher number of *α*-SMA-positive myofibroblasts, and greater infiltration of inflammatory cells. All these effects are prevented by both acylated and unacylated ghrelin [89]. Taken together, these data suggest that restoring normal circulating acylated and unacylated levels might efficiently contrast the fibrosis induced by systemic sclerosis.

## 3. Conclusions

Fibrosis is an intrinsic response to chronic injury, maintaining organ integrity when extensive necrosis or apoptosis occurs. With protracted damage, fibrosis can progress towards excessive scarring and organ failure. To date, no satisfactory treatments are available. Anti-inflammation strategies are one of the possible therapeutic approaches to fibrosis. Acylated ghrelin has a potent anti-inflammatory activity and its ability to inhibit proinflammatory cytokines expression and release has been demonstrated by a large number of studies, both in vitro and in vivo [15]. Most of the studies on the antifibrotic effects of acylated and unacylated ghrelin agree that the mechanism of action includes the reduction of inflammation. However, also their effect on oxidative stress reduction plays a crucial role in repressing the formation of fibrosis, and their broad antiapoptotic activity surely contributes in maintaining organ structure and function. This has, however, raised a doubt that if they inhibit apoptosis also in myofibroblasts, this could help, instead of hinder, fibrosis [67].

Circulating levels of ghrelin are often altered in pathologies characterized by the presence of fibrosis; however, it is difficult to discern a causative effect between ghrelin levels and fibrosis, as it is plausible that alterations in ghrelin levels reflect body mass and/or body energy metabolism. This is particularly possible in pathologies co-occurring with cachexia, such as heart and renal failure, in which the increase of ghrelin may represent a compensatory mechanism of the organism in the attempt at re-establishing optimal energetic balance or the establishment of ghrelin resistance [42]. However, in pathologies such as scleroderma, in which fibrosis affects the gastrointestinal tract, it cannot be excluded that the altered levels of ghrelin are a direct consequence of the altered gut condition.

Based on the studies reviewed herein, ghrelin, both in its acylated and unacylated forms, acts at least at two different levels. On one side, ghrelin peptides reduce the infiltration of inflammatory cells in the injured tissue and the subsequent release of cytokines responsible for fibroblast activation. On the other side, they directly affect fibroblast activity by reducing collagen production through the inhibition of TGF-*β* signaling pathway.

In conclusion, ghrelin peptides and their analogues appear to be promising in the treatment of fibrosis, although their safety and efficacy in long-term use still need to be elucidated.

## Figures and Tables

**Table 1:** Changes of acylated ghrelin, unacylated ghrelin, and obestatin blood concentrations in human pathological conditions leading to organ fibrosis.

Pathological condition	Acylated ghrelin	Unacylated ghrelin	Obestatin	Notes	Reference
Chronic heart failure (CHF)	↑	nd	nd		[46]
	↓	nd	nd	Acylated ghrelin levels positively correlate with favorable prognosis	[47]

Chronic hepatitis C	↓	nd	nd	Acylated ghrelin levels negatively correlate with fibrosis severity	[48]

Alcoholic hepatitis	↓	nd	nd	Acylated ghrelin levels negatively correlate with fibrosis severity	[48]

Nonalcoholic fatty liver disease (NAFLD)	nd	nd	=		[90]

Nonalcoholic steatohepatitis (NASH)	=	↑	=	NASH versus non-NASH (among NAFLD patients)	[50]
	↑	=	↑	Severe NASH (fibrosis index ≥2) versus not severe NASH (fibrosis index <2)	

Chronic obstructive pulmonary disease (COPD)	↑	nd	nd	Acylated ghrelin levels positively correlate with inflammation	[51]

Systemic sclerosis	↓	↓	nd		[52]

## Abbreviations

AMPK: Adenosine monophosphate-activated protein kinase
AngII: Angiotensin-II
BDL: Bile duct ligation
CLP: Cecal ligation and puncture
CTGF: Connective tissue growth factor
ECM: Extracellular matrix
EMT: Epithelial mesenchymal transition
ERK1/2: Extracellular signal-regulated kinase 1/2
GH: Growth hormone
GHSR-1a: Growth hormone secretagogue receptor type 1a
HSC: Hepatic stellate cells
IGF-1: Insulin-like growth factor 1
IL-1*β*: Interleukin-1 beta
IL-6: Interleukin-6
MCP-1: Monocyte chemoattractant protein-1
MI: Myocardial infarction
MMPs: Matrix metalloproteinases
NAFLD: Nonalcoholic fatty liver disease
NASH: Nonalcoholic steatohepatitis
NO: Nitric oxide
PAI-1: Plasminogen activator inhibitor-1
PDGF: Platelet-derived growth factor
PGC1*α*: Peroxisome proliferator-activated receptor gamma coactivator 1-alpha
PI3K: Phosphatidylinositol 3-kinase
RAS: Renin-angiotensin system
ROS: Reactive oxygen species
TAA: Thioacetamide
TGF-*β*: Transforming growth factor beta
TNF-*α*: Tumor necrosis factor-alpha
UCP2: Uncoupling protein 2
UUO: Unilateral ureteral obstruction
*α*-SMA: Alpha smooth muscle actin.
